# Increased expression of TET3 predicts unfavorable prognosis in patients with ovarian cancer-a bioinformatics integrative analysis

**DOI:** 10.1186/s13048-019-0575-4

**Published:** 2019-10-27

**Authors:** Tiefeng Cao, Wenwei Pan, Xiaoli Sun, Huimin Shen

**Affiliations:** 1 Department of Gynecology and Obstetrics, First Affiliated Hospital of Sun Yat­Sen University, Guangzhou, Guangdong 510070 People’s Republic of China; 2 Department of Obstetrics, Gynecology, & Reproductive Sciences, Yale University School of Medicine, New Haven, CT 06510 USA

**Keywords:** TET3, Methylation, Bioinformatics, Prognosis

## Abstract

Ovarian carcinoma is a lethal gynecological malignancy. Women with ovarian cancer (OC) are highly recurrent and typically diagnosed at late stage. Ten-eleven translocation protein 3 (TET3) belongs to the family of ten-eleven translocations (TETs) which induce DNA demethylation and gene regulation in epigenetic level by converting 5-methylcytosine (5mC) to 5-hydroxymethylcytosine (5hmC). Previous studies indicated that TET3 is overexpressed in ovarian cancer tissues. However, the clinic-pathological functions and prognostic values of TET3 remain unclear. Here we performed an integrative study to identify the role of TET3 by bioinformatics analysis. The TET3 expression in ovarian cancer was assessed with Oncomine database, and validated with TCGA and GTEx database. The correlation of TET3 gene alteration and clinic-pathological functions was addressed by integrative analysis of GEO datasets. Then we showed mainly TET3 gain and diploid but less deletion in ovarian cancer by copy number alteration (CNA) or mutation analysis with cBioPortal. Furthermore, by using Kaplan-Meier plotter (K-M plotter), we evaluated that high TET3 level was associated with poor survival in ovarian cancer patients, which was validated with analysis by PrognoScan database and gene differential analyses with TCGA and GTEx. This is the first study demonstrated that elevated expression of TET3 is associated with poor clinic-pathological functions, poor prognosis, wherein TET3, which presents epigenetic changes or methylation changes, might be served as a diagnostic marker or therapeutic target for ovarian cancer.

## Introduction

Ovarian cancer is the most lethal malignant tumors among female reproductive carcinoma. The most deaths are of patients with advanced stage, high-grade serous ovarian cancer. Despite improved diagnosis method and high initially response to treatment, high recurrence rate and high chemotherapy resistant rate are the main reason for low survival rate [1]. It is important to identify key prognosis factors and predictive biomarkers to provide evidence for effective targeting therapies and treatment decisions.

Epigenetic regulation plays an important role in carcinoma progress and chemo-resistance. DNA methylation changes are integral to all aspects of cancer genomics. Hypomethylation has been shown to be important in cancer progression [2]. TETs, including TET1, TET2, and TET3, are a newly discovered family of DNA demethylases that converts 5­methylcytosine to generate 5­hydroxymethylcytosine, which is subsequently converted to un-methylated cytosine, leading to DNA demethylation and gene activation [3–5]. The epigenetic modification mechanisms of TETs are functionally implicated in tumorigenesis. Large scale of articles reported that TETs mutations are found most commonly in lymphoma especially in T-cell lymphomas. In ovarian cancer, TET3 is reported as oncogene or tumor suppressor during tumorigenesis [6]. The expression of nuclear TETs was positively correlated with residual tumor and chemotherapeutic response in human ovarian cancer tissues. It showed that TET expression can influence the chemotherapy sensitivity [7], suggesting a potentially important role of TETs in the pathogenesis and chemotherapy sensitivity in ovarian cancer.

Due to tissue or sample heterogeneity among each independent experiment and the difference technological detection platforms, the identification of significantly expressed genes or proteins is inconsistent or discrepant in different studies. Thus, integration analysis with an unbiased approach should be performed [8, 9]. Therefore, we use meta-analysis in Oncomine platform to assess TET3 gene expression in ovarian cancer [10], followed by integration analysis using a larger sample size including 14 studies in GEO datasets [11], and further validated with gene expression from TCGA and GTEx. Furthermore, the survival was assessed by Kaplan-Meier plotter and validated by PrognoScan database [12, 13]. Then we identified the regulatory mechanism of genes by STRING [14, 15], and determined if the CNAs of TET3 were correlated with cancer pathological status based on cBioPortal [16, 17]. This study firstly described the expression pattern of TET3 in ovarian cancer, and the relationship between TET3 and clinic-pathological functions based on bioinformatics.

## Results

### TET3 expression was up-regulated in ovarian cancer

**Fig. 1:**
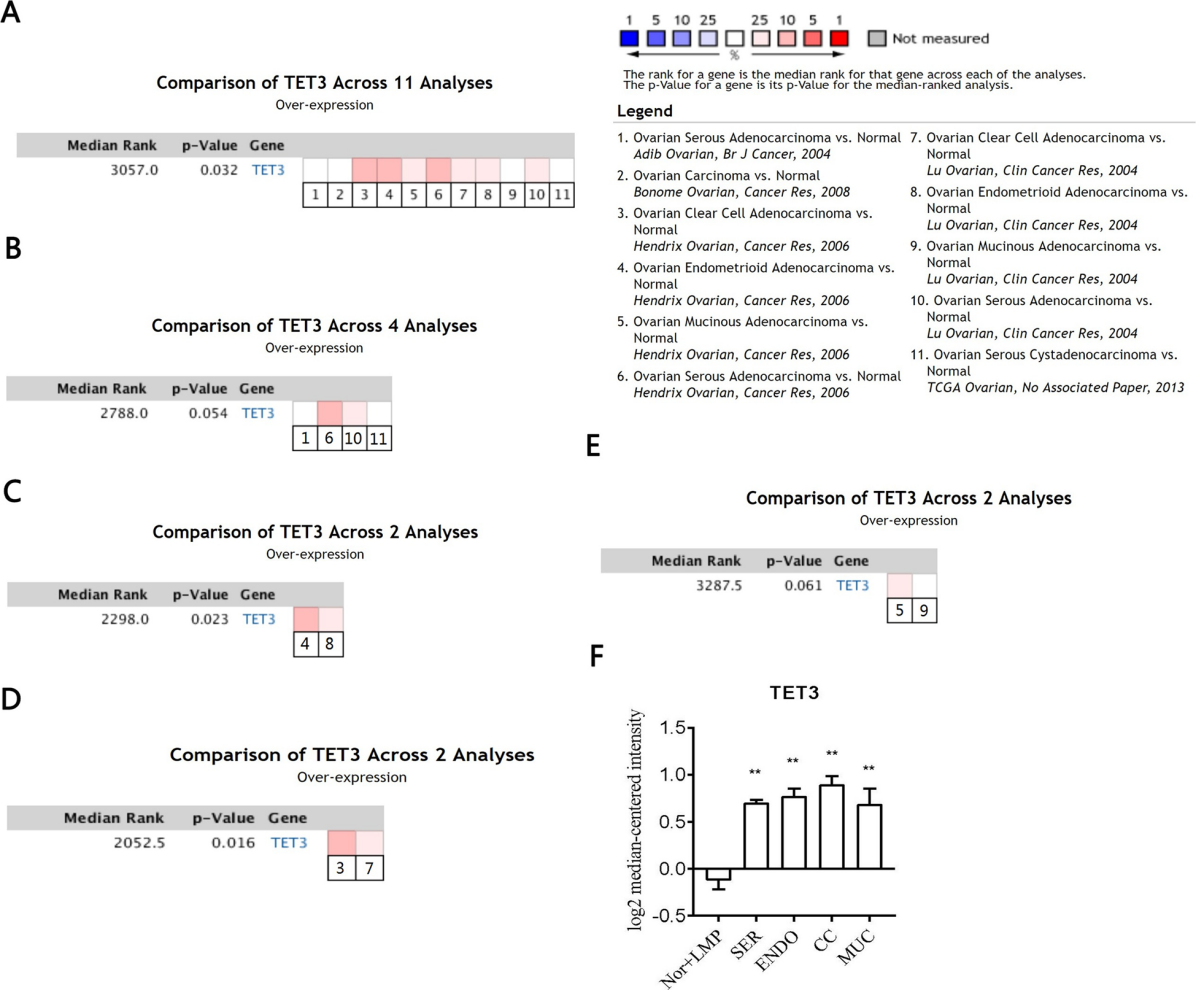
Upregulation of TET3 mRNA expression in human ovarian carcinoma by Oncomine and GEO database. TET3 is up-regulated by Oncomine meta-analysis in ovarian cancer comparing with normal tissue. Data was shown in patients with all histology (**a**); serous type (**b**); endometrioid type (**c**); with clear cell type (**d**); mucinous type (**e**). Values above the average were considered TET3 over-expression (red). TET3 mRNA expression were evaluated in 14 datasets from GEO comparing ovarian cancer in different histology vs LMP and normal tissue (**f**). Nor, normal tissue; LMP, low malignant potential tumors, or borderline tumors; SER, ovarian serous adenocarcinoma; ENDO, ovarian endometrioid adenocarcinoma; CC, ovarian clear cell adenocarcinoma; MUC, ovarian mucinous carcinoma

By the meta-analysis in Oncomine database, we analyzed TET3 gene expression levels between normal and ovarian cancer tissues in four distinct ovarian cancer datasets (Hendrix Ovarian [18]; Adib Ovarian [19]; Lu Ovarian [20]; Bonome Ovarian [21]) and TCGA ovarian dataset. The result revealed that TET3 mRNA expression levels were significantly higher in ovarian carcinoma among 11 analysis with different histology (*P* = 0.032, Fig. 1a). Besides, TET3 expression is higher in each specific histology, including serous adenocarcinoma (*P* = 0.054, Fig. 1b), endometrioid adenocarcinoma (*P* = 0.023, Fig. 1c), clear cell adenocarcinoma (*P* = 0.016, Fig. 1d), and mucinous adenocarcinoma (*P* = 0.061, Fig. 1e) in comparison with ovarian normal tissues. To verify the expression pattern of TET3 in ovarian cancer, we analyzed TET3 mRNA expression levels by integration of 14 GEO datasets (GSE18520 [22]; GSE19829 [23]; GSE23554 [24]; GSE26193 [25]; GSE27651 [26]; GSE30161 [27]; GSE32062 [28]; GSE40595 [29]; GSE44104 [30]; GSE51373 [31]; GSE54388 [32]; GSE63885 [33]; GSE65986 [34]; GSE9891 [35]) (Table 1). Comparing with borderline tumors and normal tissues, TET3 mRNA was significantly up-regulated in ovarian cancer (Fig. 1f), including serous adenocarcinoma (*P* < 0.0001, logFC = 0.811), endometrioid adenocarcinoma (*P* < 0.0001, logFC = 0.8794), clear cell adenocarcinoma (*P* < 0.0001, logFC = 1.004) and mucinous adenocarcinoma (*P* = 0.0003, logFC = 0.7978).

**Table 1:** Clinical properties of the ovarian cancer patients used in the analysis (GEO datasets)

GEO ID	Reference	GEO platform	No. of tumor samples in dataset	No. of normal samples in dataset	Death event	Median overall survival (mon)	Serous/endometrioid/clear cell/mucinous/others	Grade(I/II/III)	Stage(1/2/3/4)	Debulk optimal(/out of)	Treatment contains platin(/out of)	Treatment contains Taxol (out of)
GSE18520 [22]	Mok SC et al. 2009	GPL570	53	10	41	21	53/0/0/0/0	0/0/53	late stage^c^	NA	NA	NA
GSE19829 [23]	Konstantinopoulos PA et al. 2010	GPL570	28	0	17	35	23/0/0/0/0	0/0/28	0/2/22/4	23/27	NA	NA
GSE23554 [24]	Marchion DC et al. 2011	GPL96	28	0	14		28/0/0/0/0	2/8/18	advanced^c^	13	28	NA
GSE26193 [25]	Mateescu B et al.2011	GPL570	107	0	31	36.2	79/8/6/8/6	7/33/67	20/11/59/17	NA	NA	NA
GSE27651 [26]	King ER et al. 2011	GPL570	35	6normal 8LMP^a^	NA	NA	35/0/0/0/0	13low22high^b^	NA	NA	NA	NA
GSE30161 [27]	Ferriss JS et al. 2012	GPL570	58	0	36	33.1	47/5/1/1/2	2/19/33	0/0/53/5	30/56	58/58	54/58
GSE32062 [28]	Yoshihara K et al.2012	GPL570	10	0	NA	NA	10/0/0/0/0	0/0/10	late stage^c^	NA	NA	NA
GSE40595 [29]	Yeung TL et al.2013	GPL570	63	14	NA	NA	63/0/0/0/0	0/0/63	NA	NA	NA	NA
GSE44104 [30]	Wu YH et al. 2014	GPL570	60	0	16	NA	28/11/12/9/0	NA	17/8/30/5	NA	NA	NA
GSE51373 [31]	Koti M et al.2013	GPL570	28	0	NA	NA	28/0/0/0/0	high^b^	0/5/19/3	NA	NA	NA
GSE54388 [32]	Yeung TL et al.2017	GPL570	16	6	NA	NA	16/0/0/0/0	high^b^	NA	NA	NA	NA
GSE63885 [33]	Lisowska KM et al. 2014	GPL570	101	0	66	36.3	73/12/9/0/7	0/10/51	0/2/64/11	NA	75/75	41/75
GSE65986 [34]	Uehara Y et al.2015	GPL570	55	0	6	NA	16/14/25/0/0	NA	30/5/11/9	NA	NA	NA
GSE9891 [35]	Tothill RW et al. 2008	GPL570	267	18LMP^a^	NA	NA	264/20/0/0/1	20/99/161	24/18/217/22	NA	NA	NA

*NA* Data not available, */out of* Total number of patients with available clinical data

^a^*LMP* Low malignant potential tumors or borderline tumors

^b^No specific grade shown

^c^No specific stage shown

### High TET3 expression was correlated with poor clinicopathological features in serous ovarian cancer

**Fig. 2:**
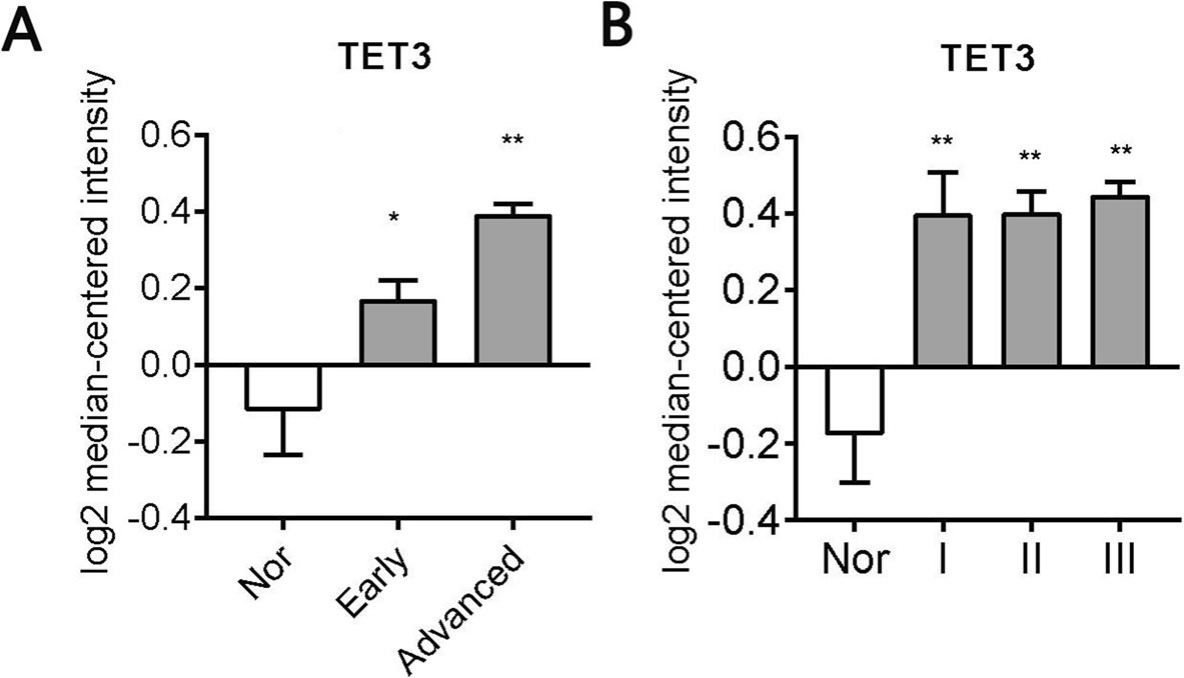
High TET3 expression was correlated with poor clinicopathological features in serous ovarian cancer. TET3 mRNA expression were evaluated in 14 datasets from GEO comparing ovarian cancer in advanced stage (III-IV) serous adenocarcinoma vs early stage (I-II) serous adenocarcinoma vs normal tissue (**a**); in Grade I, Grade II, Grade III vs normal tissue (**b**). Nor, normal tissue

Serous ovarian cancer has the highest incidence among all ovarian cancers, and high-grade serous ovarian cancer (HGSC) is the most lethal type. The correlation between TET3 expression and clinicopathologic features was analyzed to evaluate its prognostic significance of TET3 in serous ovarian cancer patients. Specially, TET3 expression is higher in serous carcinoma patients with advanced stage (III-IV) comparing with those with early stage (I-II) (*P* < 0.0001, Fig. 2a). But there is no significant difference between different grade (grade I, II, III). When comparing with normal ovarian tissues or epithelia, higher TET3 expression is shown in ovarian cancer with different histological grade (Fig. 2b), including grade I (*P* = 0.0015, logFC = 0.5655), grade II (*P* < 0.0001, logFC = 0.57) and grade III (*P* < 0.0001, logFC = 0.6147) This showed that TET3 is up-regulated in serous ovarian cancer especially those with advanced stage (III-IV).

### High TET3 expression predicts poor prognosis in ovarian cancer

**Fig. 3:**
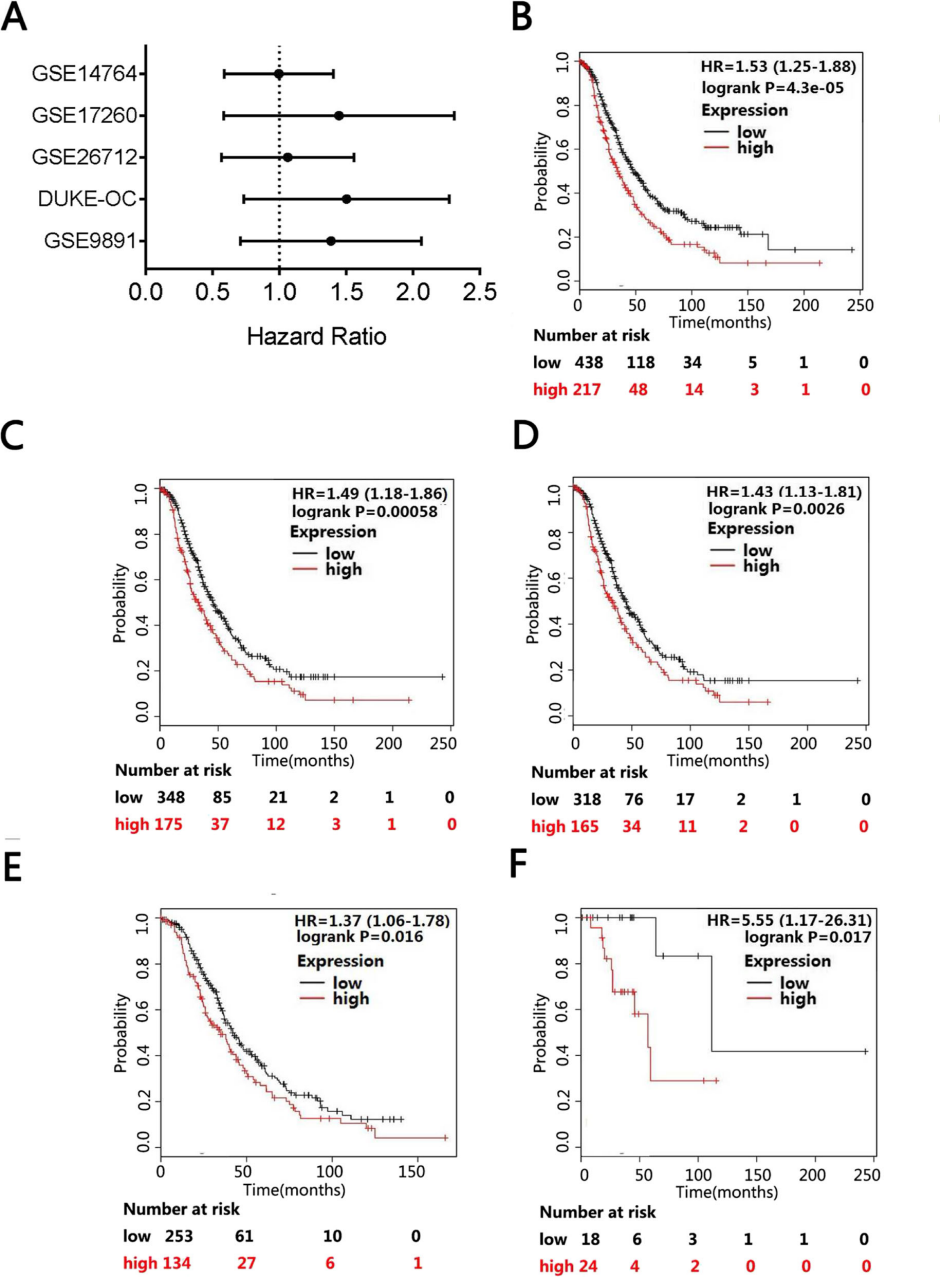
High TET3 expression predicts poor prognostis in ovarian cancer (A, PrognoScan database; B-D, K-M plotter). **a** The OS of TET3 expression in OC (PrognoScan database) is shown. The statistically significant hazard ratio in various ovarian cancer datasets was identified, and expressed as the forest plot. The analysis of survival curve was identified as the threshold of cox *p*-value < 0.05 (GSE9891, DUKE-OC, GSE26712, GSE14764). Probe ID: 214754_at. The OS survival curves of TET3 expression in ovarian cancer with all histology (*n* = 655) (**b**), serous type (*n* = 523) (**c**), high grade (II-III) serous ovarian cancer (*n* = 483) (**d**), advanced stage (III-IV) and high grade (II-III) serous ovarian cancer (*n* = 387) (**e**), early stage (I-II) and high grade (II-III) serous ovarian cancer (*n* = 42) (**f**) OS: overall survival

The prognostic significance of TET3 in ovarian cancer patients was integrated by Kaplan-Meier plotter online database and PrognoScan database. We performed meta-analysis of 5 datasets in PrognoScan database which compared OC patients with normal ovarian tissues. As shown in Fig. 3a (Additional file 1: Figure S1A), patients with higher TET3 level had significantly shorter survival time than those with a lower TET3 level. Furthermore, we evaluated the prognostic value of TET3 at mRNA level by Kaplan-Meier plotter analysis tool with cases enrolled from multiple GEO datasets and TCGA (the cancer genome atlas). It also showed that the overall survival was shorter in OC patients with higher TET3 expression (HR = 1.53 (1.25–1.88), *P* = 4.3e-05, *n* = 655, Fig. 3b), also in ovarian serous adenocarcinoma patients (HR = 1.49 (1.18–1.86), *P* = 0.00058, *n* = 523, Fig. 3c). Besides, we plotted the survival curves for the OC patients with advanced grade (II-III) serous adenocarcinoma. The overall survival was shorter in advanced patients with high TET3 expression (HR = 1.43 (1.13–1.81), *P* = 0.0026, *n* = 483, Fig. 3d). Furthermore, the relationship between TET3 expression and the overall survival in high-grade serous OC patients with different stage was evaluated. The overall survival was significant shorter in advanced stage (III-IV) patients with high TET3 expression ((HR = 1.37 (1.06–1.78), *P* = 0.016, *n* = 387, Fig. 3e), and also significant shorter in high-grade serous adenocarcinoma with early stage(I-II) (HR = 5.55 (1.17–26.31), *P* = 0.017, *n* = 42, Fig. 3f).

Higher TET3 mRNA level is also associated with poor PFS in the same way. Poor PFS was correlated with high TET3 mRNA expression level for all ovarian carcinoma patients (HR = 1.23 (1.01–1.49), *P* = 0.038, *n* = 614, Additional file 2: Figure S2A), and for serous adenocarcinoma patients (HR = 1.4 (1.13–1.74), *P* = 0.0022, *n* = 346, Additional file 2: Figure S2B). Besides, in high-grade serous adenocarcinoma, TET3 expression was significantly correlated with PFS (HR = 1.31 (1.05–1.65), *P* = 0.0179, *n* = 427, Additional file 2: Figure S2C). The PFS was significant shorter in advanced stage (III-IV) patients with high TET3 expression (HR = 1.26 (1–1.59), *P* = 0.048, *n* = 384, Additional file 2: Figure S2D), but not so significant shorter in high-grade serous adenocarcinoma with early stage (I-II) (HR =3.57 (0.8–16), *P* = 0.0765, *n* = 42, Additional file 2: Figure S2E).

### Protein components of nodes across the TET3

The protein partners in the mechanism pathway were studied with STRING database. The ten predicted protein partners of TET3 are: Protein arginine methyltransferase 5 (PRMT5), Oxoglutarate dehydrogenase (OGDH), NANOG, thymine-DNA glycosylase (TDG), DNA (cytosine-5-)-methyltransferase 1 & 3A & 3B (DNMT1 & DNMT3A & DNMT3B), Retinoblastoma binding protein 6 (RBBP6), CXXC finger protein 4 (CXXC4), YLP motif-containing protein 1 (YLPM1) with different predicted scores (Additional file 3: Figure S3A-B).

DNA methylation process is an epigenetic mechanism including methylation and demethylation. TET-DNMT complex can mediate the DNA methylation, histone modification and chromatin modification [36]. Besides, the pathway description showed that DNMTs, TDG, PRMT5 are included in chromosome modification. Combining with the predicted functional partners’ score and pathway description, we identified DNMT1, DNMT3A, DNMT3B, TDG and PRMT5 to analyze TET3.

### Analysis of TET3, TET3 related genes mutation and CNAs in cBioportal for cancer genomics database

**Fig. 4:**
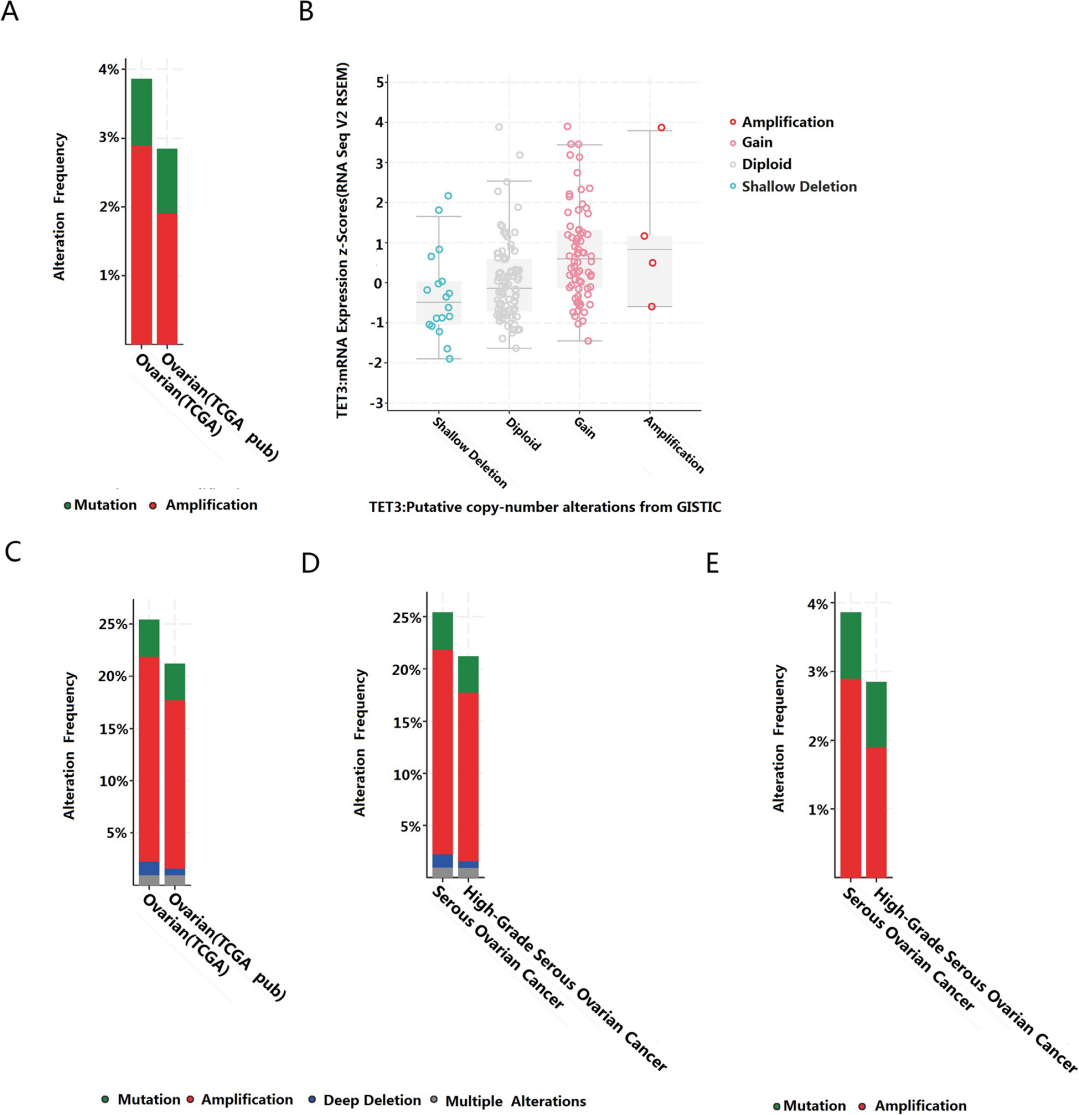
TET3, TET3 related genes mutations and CNAs in cBioportal for Cancer Genomics database. **a** and **c** The alteration frequency of TET3 gene and TET3 related genes (DNMT1, DNMT3A, DNMT3B, TDG, PRMT5 and TET3) was explored in ovarian cancers in 2 studies. **b** Relative TET3 expression mRNA level as a function of relative copy number alteration were plotted in one specific ovarian cancer database (ovarian, TCGA provisional). **d** and **e** The alteration frequency of TET3, TET3 related genes (DNMT1, DNMT3A, DNMT3B, TDG, PRMT5 and TET3) were plotted in ovarian cancer with different histology. The alteration frequency included deletions (blue), amplification (red), multiple alterations (grey) or mutation (green)

To address the correlation between TET3 mutation and cancer progression, firstly we analyzed TET3 mutation and CNAs in ovarian serous adenocarcinoma including 627 patients in 2 studies by the cBioPortal tools (ovarian: TCGA, Nature 2011 and TCGA, provisional). It is mainly TET3 amplification in ovarian carcinoma (Fig. 4a). There was more TET3 gain and diploid but less deletion in ovarian serous carcinoma. Functional plotting of TET3 indicated that TET3 mRNA expression is associated with genetic status (deletion or amplification) of TET3 in ovarian cancer (Fig. 4b).

Then, we analyzed the six genes correlated with TET3 pathway by the cBioPortal tools in these 627 samples. These 2 studies contained > 20% alteration frequency and the amplification occurred predominantly in ovarian cancer (Fig. 4c). Minor deletion or multiple alterations occurred in these datasets. The ratio of amplification or mutation is almost the same in either serous ovarian cancer or high-grade serous ovarian cancer about TET3 alone or six genes (Fig. 4d and e).

Furthermore, we used the Oncoprint for gene alteration and mRNA regulation of 6 genes in ovarian cancer (TCGA, provisional, *n* = 311). The alteration percentages is between 7 and 18% (DNMT1, 17%; DNMT3A, 11%; DNMT3B, 12%; TDG, 12%; PRMT5, 13%; TET3, 13%) (data not shown) .

### TET3 is involved in epigenetic regulation

TET3 is the demethylase and may regulate cancer progression in the epigenetic level. By using ICGC database [37], we performed GO analysis, and showed that TET3 is involved in 5-MC catabolic process, DNA demethylation and epigenetic regulation of gene expression (Additional file 4: Figure S4). The relationship of TET3 and ovarian cancer progression is understudied.

### Validation using independent microarray datasets

To confirm the repeatability and portability of TET3 expression pattern in ovarian cancer, we compared TET3 expression in ovarian cancer cases in TCGA and normal cases in GTEx database, as validation dataset of TET3 using this independent microarray data. As shown in Additional file 5: Figure S5, TET3 is up-regulated in ovarian tumors, while TET1 doesn’t change. On the contrary, Additional file 5: Figure S5 depicts down-regulation of TET2 in ovarian cancer, which is in accordance with the structure and the opposite functional value of TET2 when comparing with TET3. Further, GSE17260 and DUKE-OC in PrognoScan database validated the prognostic value of TET3 in ovarian cancer (Additional file 1: Figure S1).

## Discussion

Ovarian cancer are highly heterogeneous in terms of histopathology, treatment options, and clinical outcomes. Emerging evidence shows that genetic mutations and copy number alteration underlie the pathogenesis and heterogeneity of ovarian cancer. For example, high grade serous ovarian cancer, which is the most common and most aggressive histotype, generally shows gross copy number variations [38]. But low grade serous ovarian cancer appears to possess Ras family of genes mutations [39]. Ovarian cancer has a variety of histotypes, thus more and more work has done to identify genetic differences between histotypes, and epigenetic changes are emerged to be characterized.

The TET proteins (including TET1, TET2, and TET3) are a newly found family. TETs are DNA demethylases that act to oxidize 5-methylcytosine to generate 5-hydroxymethylcytosine, which is subsequently converted to unmethylated cytosine and leading to DNA demethylation and gene activation. They have the critical role on gene methylation and epigenetic regulation. Usually less TET3 is detected in normal ovary tissues. In current study, we observed that TET3 was upregulated in ovarian cancer tissues compared with normal controls. And we addressed that high TET3 is correlated with higher stage and poor clinicopathological features, suggesting a potentially important role of TETs in the pathogenesis of ovarian cancer.

Interestingly, Kaplan-Meier Plotter and PrognoScan database analysis showed that high level of TET3 expression results in shorter survival rate in ovarian cancers, especially in high-grade (II-III), advanced stage (III-IV) ovarian cancers (Fig. 3), indicating that high TET3 expression in OC predicts poor survival. Thus, TET3 may be an oncogene in ovarian cancer. But it is unclear that why TET3 is upregulated in ovarian cancer and what the specific mechanism is for the epigenetic regulation.

This increased expression of TET3 in OC may be partly contributed by DNA copy number amplification. TET3 CNA and gene mutation data showed that TET3 amplification or TET3 gain is the most commonly frequency in ovarian cancer, not only in the high-grade but also in low-grade serous adenocarcinoma. Subsequently, drugs inhibiting TET3 DNA amplification can rescue ovarian cancer progression and survival. But we still need more evidence to explain the upregulation of TET3.

DNA methylation (methyl CpG) is the most commonly studied epigenetic modification, through the attachment of a methyl group to the C5 position of cytosines (5mC) in a CpG context. TET3 is one kind of demethylase, and TET3 was ever thought to have effect on the methylation status’ changes. Our study demonstrated that proteins related with TET3 are mainly DNA methylase or demethylases such as DNMT, and most of these genes showed amplification in ovarian cancer. When treatment of acquired platinum-resistant ovarian cancer with guadecitabine (DNMT inhibitor) and carboplatin, TET3 expression is dramatically up-regulated (logFC = 1.04, GSE102118 [40], data not shown), indicating that TET3 can have a role with the combination of DNMT or other methylases. Despite the enormous medical and economic impact of ovarian cancers, there are few options for the OC treatments. The main feature of ovarian cancer is the chemotherapy resistant and late diagnosis. About 75% of advanced ovarian cancer patients respond to chemotherapy treatment initially, but most of themwould have the chemotherapy resistance. Han X [6, 7] showed that TET1 promotes cisplatin-resistance via demethylating the vimentin promoter in ovarian cancer. GSE1926 also compared the carboplatin sensitive and resistant primary ovarian cancer cells from patients [41], and the result showed that TET3 is dramatically up-regulated in carboplatin resistant cells (*P* = 3.26–05, logFC = 0.48, data not shown). This indicated that TET3 may play a role in carboplatin resistance and sensitivity restoration by changing methylation status. Our identification of TET3 as a prognostic factor and maybe a chemotherapy sensitive marker suggests a potentially unifying clinical role of TET3. It will be fascinating to test in the future whether targeting TET3 leads to new treatment options of ovarian cancer especially platinum resistance ovarian cancer.

In conclusion, here we firstly indicated the role of TET3 in ovarian cancer progression. By the interpretation of all the oncogenic data with the bioinformatics, we addressed the molecular mechanisms related with methylation in ovarian cancer.

## Materials and methods

### Gene expression omnibus (GEO) database

The GEO database (http://www.ncbi. nlm.nih.gov/geo) is a public data storage repository, mainly the microarray, methylation and next-generation sequencing data. We identified 3 datasets including 18 profiles. For the gene expression dataset, by searching “ovarian cancer”, “gp196”,“gp570” and “gp571”, 14 profiles associated with gene expression data, clinical and pathological information were included. For this analysis, we only used three microassay platforms because they are frequently used and the three platforms are almost identical and probe sets are identical to avoid the varying accuracy with different relative scales and with diverse dynamic ranges [8]. We download raw. CEL files of these microarray gene expression data, normalized with MAS5 in the R statistical environment (www.r-project.org) using the affy Bioconductor library, second normalized by setting the average expression on each chip to 1000 to reduce batch effects [9], and then make the interpretation with all these 14 profiles data.

### TCGA and GTEx gene expression data

To solve the imbalance between the tumor and normal data which can cause heterogeneity in differential analyses, we downloaded the TCGA and GTEx (Genotype-Tissue Expression) gene expression data re-computed from raw RNA-Seq data by the UCSC Xena project, normalized in the R statistical environment using the affy Bioconductor library NormalizeBetweenArrays.

### Oncomine database analysis

To know the expression level of TET3 in ovarian cancer, Oncomine database (https://www.oncomine.org) was used. Oncomine is a public online database consisting of previously published and publically available microarray data [10]. The analysis of mRNA expression fold change was filtered by selecting ovarian carcinoma vs normal analysis. The standardized normalization and parameters are provided on the Oncomine platform as follows: *p*-value<1E-4, fold change > 2, and gene ranking in the top 10%.

### cBioportal database

The cBioPortal (http://cbioportal.org) is an open access resource which provides visualization and analysis for cancer genomics data sets including copy number variation and mutation. This portal provided access data derived from 147 individual cancer studies of 31 types of cancer and over 21,000 samples [16]. The search cancer includedovarian serous cystadencarcinoma and the search parameters included alterations, CNA and RNA seq data.

### Kaplan-Meier (KM) plotter database

The Kaplan Meier plotter (http://kmplot.com/analysis) is based on an online database [12] and is capable to assess the association of genes on survival in four types of cancer samples including breast cancer, ovarian cancer, lung cancer and gastric cancer. Clinical data included gender, age, histology, stage, grade, TP53 mutation status and applied chemotherapy for all patients. The correlation of individual TET3 mRNA expression and survival in ovarian cancer was analyzed online and presented with the hazard ratio, 95% confidence intervals and computed log rank *p*-value.

### PrognoScan database

PrognoScan database (http://www.abren.net/PrognoScan/) includes a large-scale collection of publicly available cancer microarray datasets with clinical information and can be used to analysis the biological relationship between gene expression and prognosis [13]. We used PrognoScan database to identify the correlation between TET3 mRNA expression and survival in serous ovarian cancer with the adjusted cox *p*-value < 0.05.

### STRING analysis

The STRING analysis (http://string-db.org) is an online tool to analysis the protein-protein interaction and functional protein networks. To identify the TET3-related proteins, we used TET3 as the query. Several proteins were verified based on the predicted functional partners’ score and the biological process analysis mainly in chromatin modification and DNA methylation. Those with low predicted functional partners’ score and not specific to DNA modification or methylation were excluded from the gene signature.

### Statistical analysis

Download data was analyzed using GraphPad Prism version 7 (GraphPad Software, La Jolla, CA, USA). GEO data and Oncomine figures were explored with online tools. Survival curves were generated with Kaplan-Meier plots and PrognoScan online tools. All results are presented with *P* values from a log-rank test. Statistical significance of the data (*P*-values) was provided by the program.

## Conclusions

In summary, this study used bioinformatics analyses by different database and revealed that TET3 is correlated with cancer progression, prognosis. TET3 is demethylase and related to epigenetic modification, so TET3 might be a good target for cancer treatment.

## Supplementary information

**Additional file 1: Figure S1.** The prognostic effect of TET3 mRNA expression in ovarian cancer (PrognoScan database). The Kaplan-Meier plot from PrognoScan database of high or low level TET3 mRNA expression in ovarian cancer are from three typical datasets with cox *p*-value < 0.05, including GSE9891 (A); GSE17260(B); DUKE-OV (C). Probe ID: 214754_at.

**Additional file 2: Figure S2.** High TET3 expression predicts poor PFS in ovarian cancer (K-M plotter). (A) The PFS survival curves of TET3 expression in ovarian cancer with all histology (*n* = 614) (B), serous type (*n* = 483) (C), high grade (II-III) serous ovarian cancer (*n* = 427)(D), advanced stage (III-IV) and high grade (II-III) serous ovarian cancer (*n* = 384) (E), early stage (I-II) and high grade (II-III) serous ovarian cancer (*n* = 42) (F).

**Additional file 3: Figure S3.** Protein components of nodes across TET3. Colored nodes are the proteins related with TET3 by using String, v10.5 (http://string-db.org). (A) Predicted functional partners of TET3 are shown based on published data and database. (B) Predicted functional partners with different score are shown.

**Additional file 4: Figure S4.** The potential pathway and GO processes were visualized by ICGC Data Portal with TET3.

**Additional file 5: Figure S5.** Validation of TETs expression with TCGA and GTEx. Gene expression of TET1/TET2/TET3 is shown when comparing ovarian tumor cases in TCGA and norma ovarian tissues in GTEx. TCGA, the cancer genome atlas; GTEx, Genotype-Tissue Expression.

## Data Availability

The datasets generated and analysed during the current study are available from TCGA, GEO, Oncomine, Kaplan-Meier plotter, PrognoScan, cBioPortal data, STRING analysis and ICGC that provide free online tools and resources.
