# Supplementary figures and images for: Increased expression of TET3 predicts unfavorable prognosis in patients with ovarian cancer-a bioinformatics integrative analysis
Source: J Ovarian Res. 2019 Oct 27;12:101. doi: 10.1186/s13048-019-0575-4 (PMC6816171; doi:10.1186/s13048-019-0575-4)

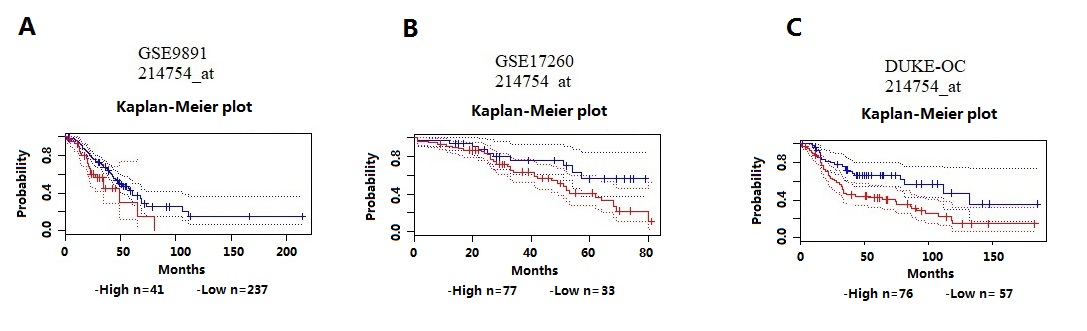

Supplement: Supplementary file 1 — Additional file 1: Figure S1. The prognostic effect of TET3 mRNA expression in ovarian cancer (PrognoScan database). The Kaplan-Meier plot from PrognoScan database of high or low level TET3 mRNA expression in ovarian cancer are from three typical datasets with cox p-value < 0.05, including GSE9891 (A); GSE17260(B); DUKE-OV (C). Probe ID: 214754_at. [file 13048_2019_575_MOESM1_ESM.jpg]

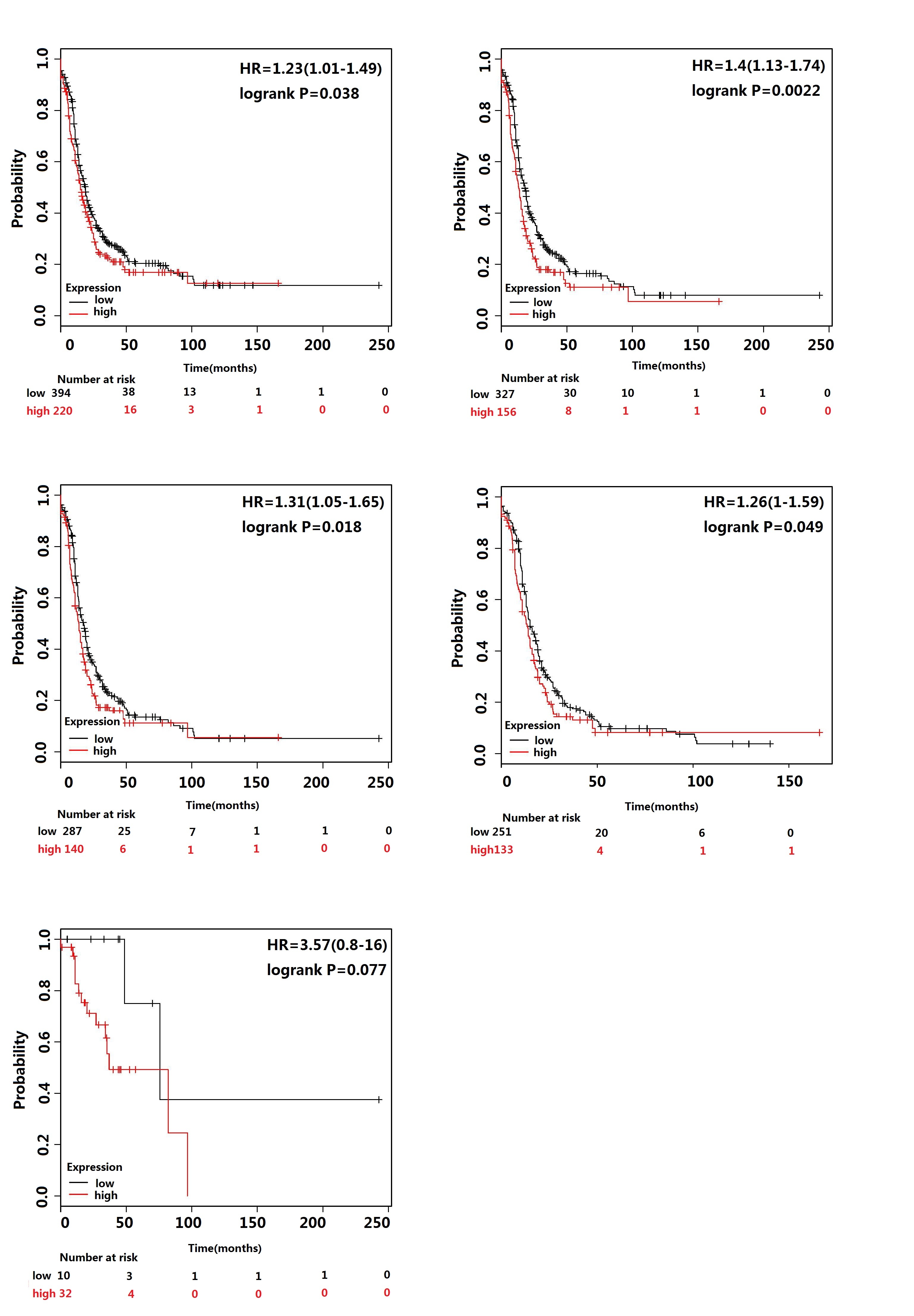

Supplement: Supplementary file 2 — Additional file 2: Figure S2. High TET3 expression predicts poor PFS in ovarian cancer (K-M plotter). (A) The PFS survival curves of TET3 expression in ovarian cancer with all histology (n = 614) (B), serous type (n = 483) (C), high grade (II-III) serous ovarian cancer (n = 427)(D), advanced stage (III-IV) and high grade (II-III) serous ovarian cancer (n = 384) (E), early stage (I-II) and high grade (II-III) serous ovarian cancer (n = 42) (F). [file 13048_2019_575_MOESM2_ESM.jpg]

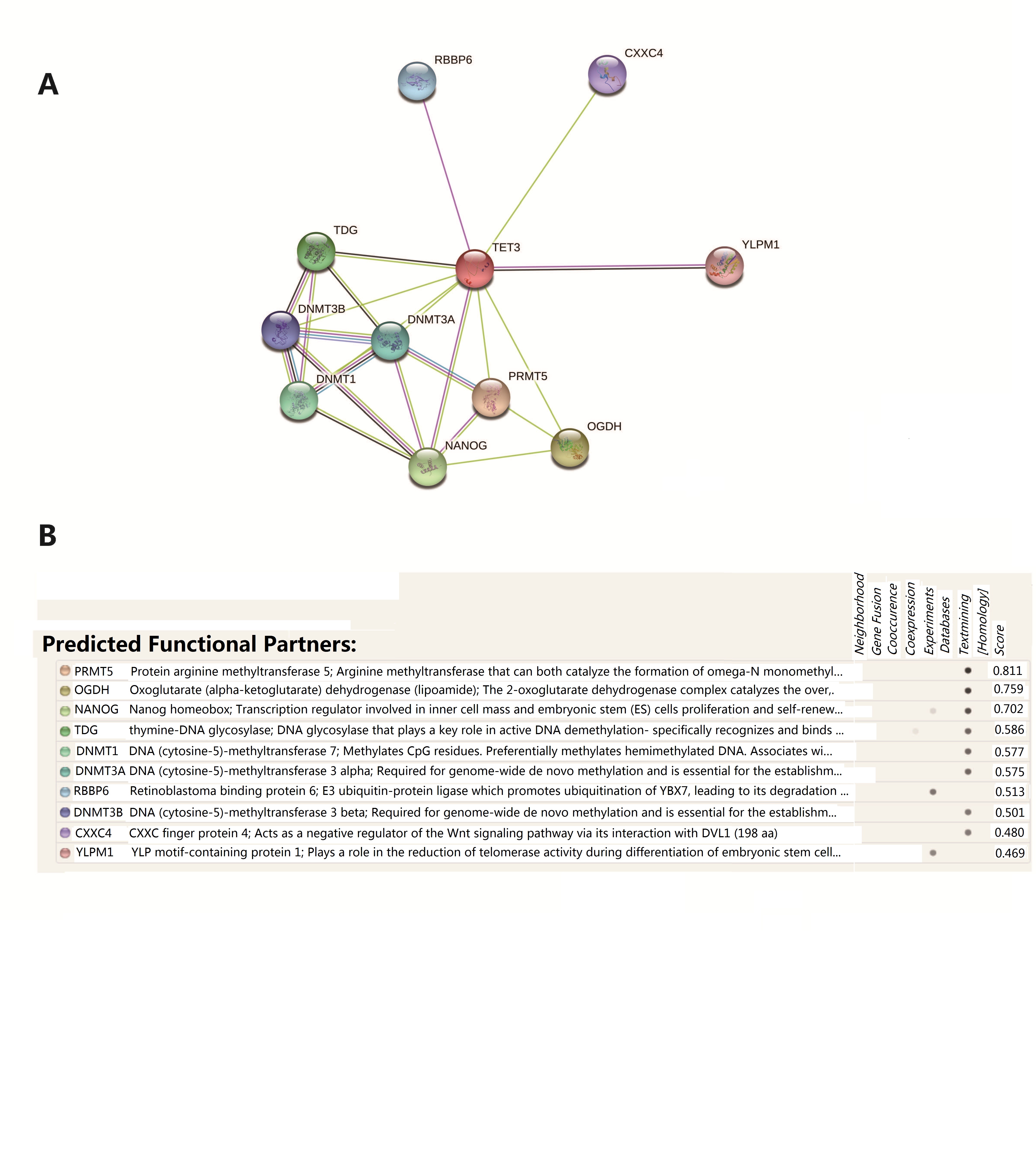

Supplement: Supplementary file 3 — Additional file 3: Figure S3. Protein components of nodes across TET3. Colored nodes are the proteins related with TET3 by using String, v10.5 (http://string-db.org). (A) Predicted functional partners of TET3 are shown based on published data and database. (B) Predicted functional partners with different score are shown. [file 13048_2019_575_MOESM3_ESM.jpg]

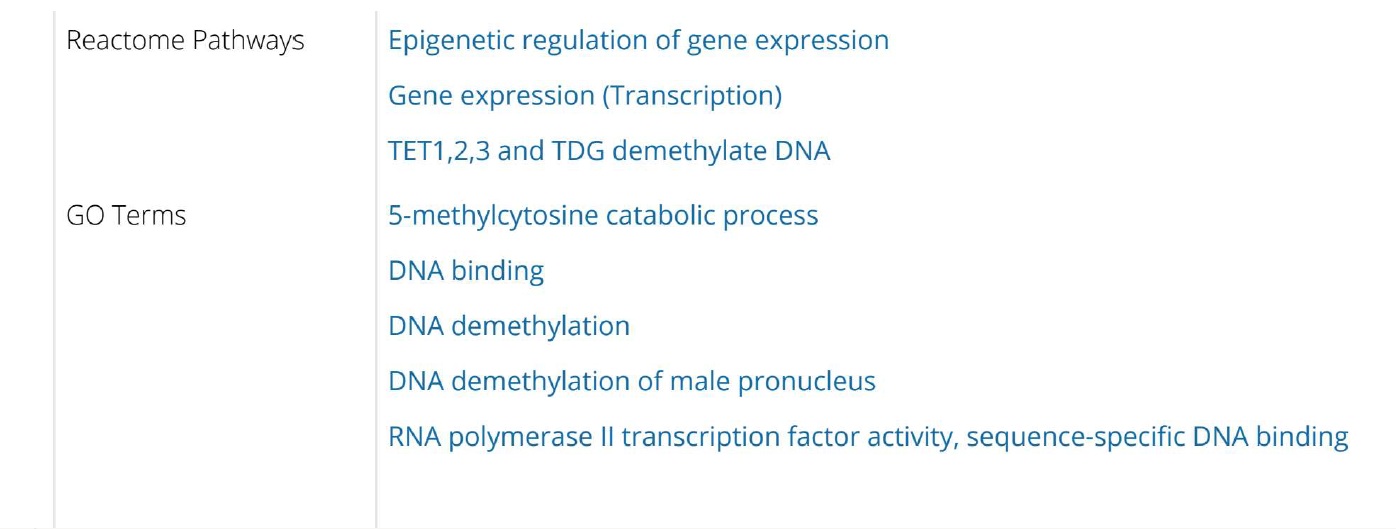

Supplement: Supplementary file 4 — Additional file 4: Figure S4. The potential pathway and GO processes were visualized by ICGC Data Portal with TET3. [file 13048_2019_575_MOESM4_ESM.jpg]

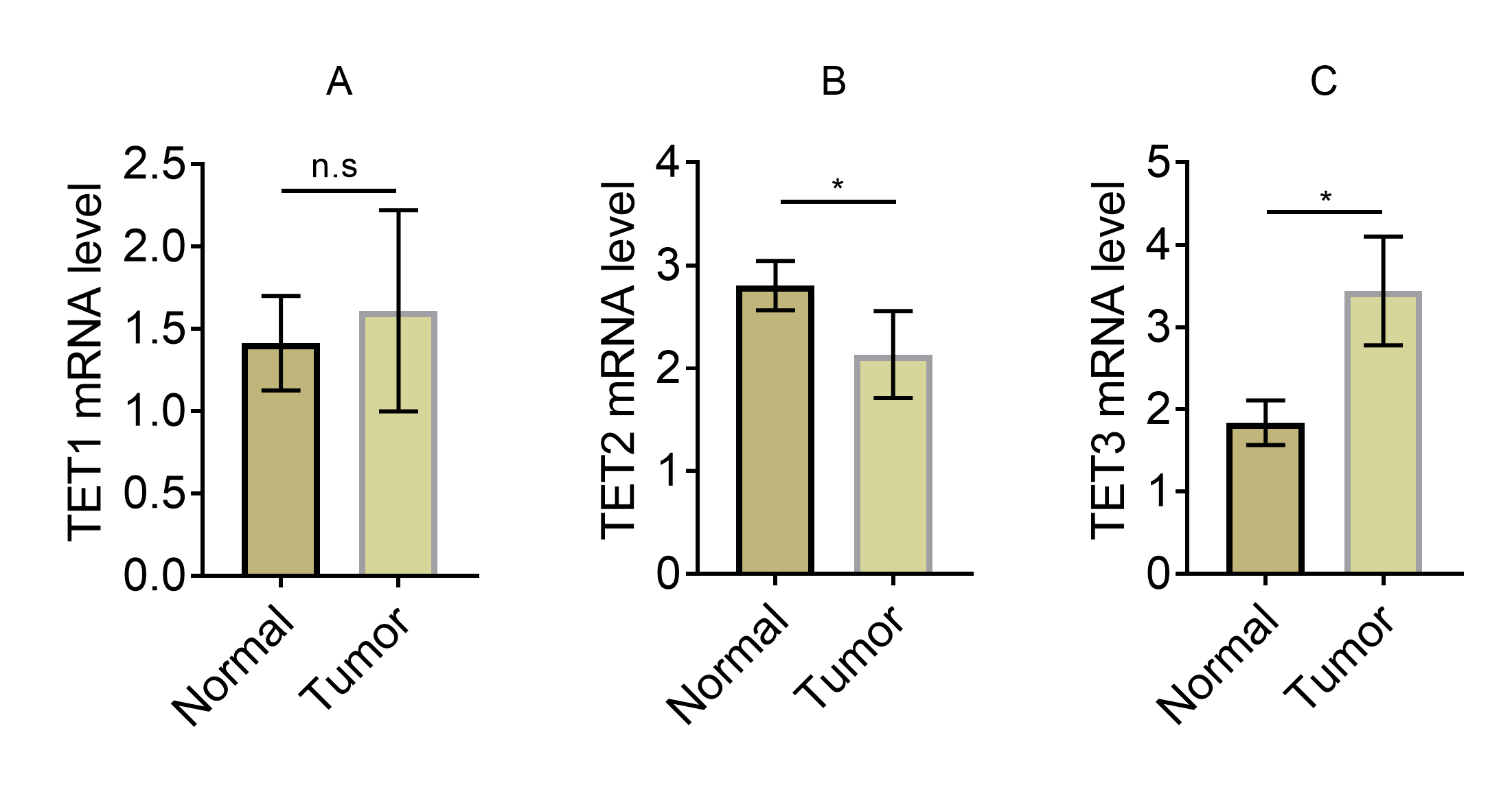

Supplement: Supplementary file 5 — Additional file 5: Figure S5. Validation of TETs expression with TCGA and GTEx. Gene expression of TET1/TET2/TET3 is shown when comparing ovarian tumor cases in TCGA and norma ovarian tissues in GTEx. TCGA, the cancer genome atlas; GTEx, Genotype-Tissue Expression. [file 13048_2019_575_MOESM5_ESM.tif]
